# Coinheritance of non‐deletional hemoglobin H disease with sickle cell trait

**DOI:** 10.1002/jha2.916

**Published:** 2024-05-24

**Authors:** Veroniki Komninaka, Evangelia‐Eleni Ntelaki

**Affiliations:** ^1^ Centre of Excellence in Rare Hematological (Hemoglobinopathies) and Rare Metabolic (Gaucher Disease) Diseases Laiko General Hospital Athens Greece

**Keywords:** coinheritance, Hemoglobin H, sickle cell trait

1

There are limited reports in the literature about the coinheritance of deletional hemoglobin H (HbH) disease and hemoglobin S (HbS) heterozygosity and even more limited ones about non‐deletional HbH disease and sickle cell trait [1]. We present a 51‐year‐old male of Greek origin who was referred to the clinic for anemia, splenomegaly, and fatigue investigation. The lab results were: red blood cells (RBC) 6.82 × 10^12^/L, Hb 95 g/L, hematocrit 0.35, mean corpuscular volume 55.9 fL, mean corpuscular hemoglobin 1.85 fmol, mean corpuscular hemoglobin concentration 18.5 mmol/L, red cell distribution width 0.279, RET 1.55%, white blood cells 6.2× 10^9^/L, PLT 350 × 10^9^/L, serum ferritin 0.05 nmol/L (reference values 0.04–0.4), serum Fe 13.6 nmol/L (reference values 11–22), and reduced erythrocyte life span T1/2^51^Cr 14 days. On the blood smear, we found anisocytosis, poikilocytosis, anisochromia, microcytosis, and target cells (Figure 1A). Brilliant cresyl blue RBC staining showed typical HbH inclusions (Figure 1B). The sickle cell test (with sodium metabisulphite) was positive. High‐performance liquid chromatography showed HbA 70.7%, HbS 18.5%, HbA_2_ 3.0%, HbF 0.4%, and persistence of HbH/Hb Bart's 5.2/2.8% (Figure 2A). HbA_2_ with DEAE chromatography was 1.7%. Genetic analysis using Southern blotting for the beta‐globin gene and Southern blotting for the alpha globin locus revealed HbS trait (HBB:c.20A > T) and homozygosity for a point mutation in the alpha 2 globin gene polyadenylation signal (HBA2:c.*94A > G (these findings were confirmed over time using Sanger sequencing). The radiology tests revealed splenomegaly 20 × 10 × 11 cm and osteoporosis, but no extramedullary masses, and endoscopy for gastrointestinal bleeding was negative. The patient was diagnosed with a rare combination of non‐deletional HbH disease and sickle cell trait.

**FIGURE 1:**
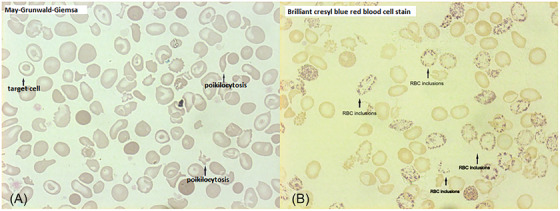
(A) Blood smear morphology (B) RBC inclusions.

**FIGURE 2:**
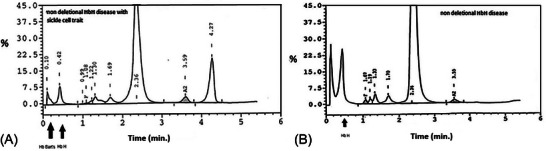
(A) HbH HbS HPLC (B) HbH HPLC.

It is known that α‐thalassemia with sickle cell trait results in a lower than usual percentage of HbS, a finding also detected in our patient (HbS 18.5%), who experienced no pain crisis. On the other hand, when HbH disease is inherited with sickle cell trait (β‐gene mutation) may produce hemolytic anemia with decreased or non‐detectable β‐globin tetramers HbH. The HbH percentage of our patient was 5.2% in contrast with higher HbH levels, approximately 20%–30%, in patients with HbH disease without the coinheritance of the HbS trait (Figure 2B), while in the study of Al Moamen et al. HbH ranged from 7.5% to 27.5% in the specific alpha genotype [2]. The reason is that there are decreased β^A^‐globin chains available for HbH formation since only the non‐mutant β‐globin gene synthesizes these chains. Additionally, α‐globin chains have a greater affinity for β^A^ than for β^S^‐chains, forming HbA leading to excess free β^S^‐chains. Under these terms, the lower‐than‐expected percentage of HbS in our patient is explained by the different affinity of a‐chains for β^A^ and β^S^‐chains [3, 4].

αΤSaudi homozygosity is linked to HbH clinical phenotype, despite that only two out of four α‐genes are affected. The severity of the specific mutation is proposed to be due to a transcriptional interference mechanism leading to the structurally normal α1‐globin gene downregulation [2]. The above‐mentioned lower HbH quantity could lead to a milder type of HbH disease with less ineffective hematopoiesis and therefore reduced iron overload (our patient had no iron overload) [5].

## AUTHOR CONTRIBUTIONS

Conceptualization: Veroniki Komninaka; Methodology and data collection: Veroniki Komninaka and Evangelia‐Eleni Ntelaki, Supervision: Veroniki Komninaka; Writing—original draft preparation: Veroniki Komninaka; Writing—review and editing: Veroniki Komninaka. All authors have read and agreed to the published version of the manuscript.

## CONFLICT OF INTEREST STATEMENT

The authors declare no conflict of interest.

## FUNDING INFORMATION

This manuscript received no external funding.

## ETHICS STATEMENT

The authors have confirmed ethical approval statement is not needed for this submission.

## PATIENT CONSENT STATEMENT

All participants in this study signed consent forms.

## CLINICAL TRIAL REGISTRATION

The authors have confirmed clinical trial registration is not needed for this submission.

## Data Availability

The data presented in the manuscript are available from the corresponding author upon reasonable request.
